# DOT-AE-GAN: a hybrid autoencoder–GAN model for enhanced ultrasound-guided diffuse optical tomography reconstruction

**DOI:** 10.1117/1.JBO.30.7.076003

**Published:** 2025-07-03

**Authors:** Md. Iqbal Hossain, Minghao Xue, Lukai Wang, Quing Zhu

**Affiliations:** a Washington University, Imaging Science, St. Louis, Missouri, United States; b Washington University School of Medicine, Department of Biomedical Engineering, St. Louis, Missouri, United States; c Washington University School of Medicine, Department of Radiology, St. Louis, Missouri, United States

**Keywords:** breast cancers, diffuse optical tomography, ultrasound-guided diffuse optical tomography, autoencoders, generative adversarial networks, deep learning

## Abstract

**Significance:**

Diffuse optical tomography (DOT) is a noninvasive functional imaging technique; however, the reconstruction of high-quality images from DOT data is a challenging task because of the ill-posed nature of the inverse problem. We introduce a hybrid machine learning model that combines the strengths of autoencoders (AEs) and generative adversarial networks (GANs) for robust DOT reconstruction.

**Aim:**

We leveraged a hybrid machine learning model for robust ultrasound-guided DOT reconstruction.

**Approach:**

A hybrid model, DOT-AE-GAN, that combines the strengths of AEs and GANs to enhance the robustness of DOT reconstruction is introduced. The proposed model utilizes an AE to efficiently encode the DOT measurement to reconstruction and decode back to measurement, modeling the inverse and forward process of reconstruction. In parallel, a GAN framework is incorporated to enhance the robustness of the reconstruction for irregularly shaped lesions, utilizing adversarial training.

**Results:**

The DOT-AE-GAN model is first trained and validated using simulations, demonstrating reconstruction accuracy in absorption coefficients and lateral dimensions of the targets. The DOT-AE-GAN is then fine-tuned with phantom data and compared with the AE model, showing the improvement over the AE model in the reconstructed target lateral dimension while keeping similar accuracy in absorption coefficient. The DOT-AE-GAN is validated with patient data, revealing that the DOT-AE-GAN-reconstructed breast lesion lateral dimensions follow size measurements of co-registered ultrasound significantly better than the optimization-based reconstruction algorithm and AE model with improved absorption contrast between malignant and benign lesions.

**Conclusions:**

Our results demonstrate that the DOT-AE-GAN model has great potential in ultrasound-guided DOT reconstruction.

## 1 Introduction

Breast cancer is the most common invasive cancer among women in the United States, accounting for ∼30% of all new female cancer cases each year, and it poses a critical global health concern that significantly impacts both physical health and emotional well-being, affecting countless individuals and families.^1^ The American Cancer Society estimates that breast cancer will be the most commonly diagnosed cancer among women, with more than 310,720 new cases and ∼42,250 deaths expected in the United States for the year 2024.^2^ Therefore, the diagnosis and prognosis of breast cancer are crucial in the battle against this formidable disease. Various imaging modalities, such as X-ray mammography, ultrasound, and magnetic resonance imaging, are widely used for the detection and diagnosis of breast cancer.^3^^–^^6^ However, X-ray mammography and ultrasound are not functional imaging modalities. In contrast, although magnetic resonance imaging is a functional imaging technique, it is expensive and only recommended for patients with a high risk of breast cancer.^7^ Diffuse optical tomography (DOT) could be a potential alternative due to its noninvasive nature and its ability to provide functional information about biological tissues.^8^ By utilizing near-infrared light, DOT can measure the optical properties of biological tissue, which are indicative of tissue composition and function. This technique has found applications in many fields, including breast cancer detection, brain imaging, and muscle disease monitoring. Despite its potential, DOT faces challenges in image reconstruction, primarily due to the intensive light scattering in tissue which causes the ill-posed nature of the inverse process.

The reconstruction of DOT involves solving an inverse problem where the goal is to recover the internal distribution of optical properties from boundary measurements of scattered light. This process is complicated by the fact that light propagation in biological tissue is highly scattered, making direct inversion difficult. Again, localizing the target lesion with DOT alone is highly challenging. To assist in the localization of the target lesion, an ultrasound probe is integrated alongside the DOT probe in the ultrasound-guided DOT system.^9^ In this system, the co-registered ultrasound (US) images are useful to provide structural information on the target lesion, which is later used for DOT reconstruction from the measured DOT data.

Traditional reconstruction methods, such as iterative algorithms and Tikhonov regularization,^10^ often struggle to produce high-quality images due to their sensitivity to noise in the ill-conditioned inverse process. Recent advances in machine learning have opened new avenues for tackling such complex inverse problems in imaging.^11^^–^^14^ Some deep learning approaches have been developed on the foundation of iterative methods, leveraging image priors to enhance or denoise DOT-reconstructed images.^15^^–^^19^ Abhishek et al.^20^ used geometric prior for DOT reconstruction. In addition, certain deep learning models have adopted end-to-end architectures for reconstruction directly from DOT measurements.^14^^,^^21^ However, these models have focused on the reconstruction of simple targets.

Autoencoders (AEs)^19^ are a class of neural networks that learn efficient data representations by compressing input into a latent space and then reconstructing it from this compressed form. In our framework, this encoding–decoding structure aligns well with the inverse problem in DOT: the encoder learns to map measurement data to reconstructed optical absorption maps, whereas the decoder approximates the forward photon transport process by mapping the reconstructions back to the perturbation space. Training the autoencoder end-to-end allows the network to learn both inverse and forward mappings in a physically consistent manner. This architecture is especially advantageous for ill-posed problems such as DOT, where multiple solutions can correspond to the same set of measurements. The latent representation learned by the autoencoder serves as an implicit prior, guiding the solution toward more plausible reconstructions and improving robustness against noise and ambiguity. Zou et al.^14^ leveraged this property of autoencoder and designed a physics-constrained DOT reconstruction model. However, although traditional feed-forward neural networks excel at modeling complex functions, they rely heavily on the type of training data. These networks perform exceptionally well when trained on data that comprehensively covers all potential output domains; however, their performance significantly deteriorates when faced with data outside this domain. Simulating breast lesions with varying complex shapes presents significant challenges. Simple shapes, such as spheres or ellipses, are more straightforward to model, but training a network on this limited data constrains its ability to generalize the model to the diverse and irregularly shaped lesions often encountered in patients’ data. Conversely, generative neural networks (GANs),^22^ which consist of generator and discriminator networks trained through adversarial learning, enable more diverse training. This adversarial process can be leveraged to train the autoencoder model^19^ in reverse—from the decoder to the encoder—enhancing the network’s robustness in reconstructing lesions of varying shapes.

In this paper, we propose DOT-AE-GAN, a hybrid model that combines the strengths of autoencoders and GANs to address the challenges of ultrasound-guided DOT image reconstruction. The autoencoder component of DOT-AE-GAN is employed to encode DOT-measured data to reconstruction and to decode back to DOT-measured data from the reconstruction. Concurrently, to make the reconstructions more robust for diverse lesion shapes, we incorporated GAN, which makes the autoencoder trainable from decoder to encoder.

The structure of this paper is as follows. In Sec. 2, we provide an overview of our DOT device. Section 3 describes the architecture and implementation details of the proposed DOT-AE-GAN model. In Sec. 4, we outline the details of the training process and the associated loss functions. In Secs. 5 and 6, we present the experimental setup, datasets used, evaluation metrics, and results. Finally, Sec. 7 concludes the article and outlines the potential directions for future research.

## 2 DOT System and Data Acquisition

Photon transport in biological tissue can be effectively modeled using the diffusion equation, which describes the behavior of light as it propagates through a highly scattering medium, where photons undergo multiple scattering events. Image reconstruction in DOT involves the forward and the inverse problems. The forward problem usually uses the diffusion equation to predict the distribution of the measurements based on presumed parameters for both the light source, boundary conditions, and the object. The forward process in a semi-infinite homogeneous medium can be linearized with the Born approximation. The inverse process typically reconstructs the object using optimization approaches.

**Fig. 1:**
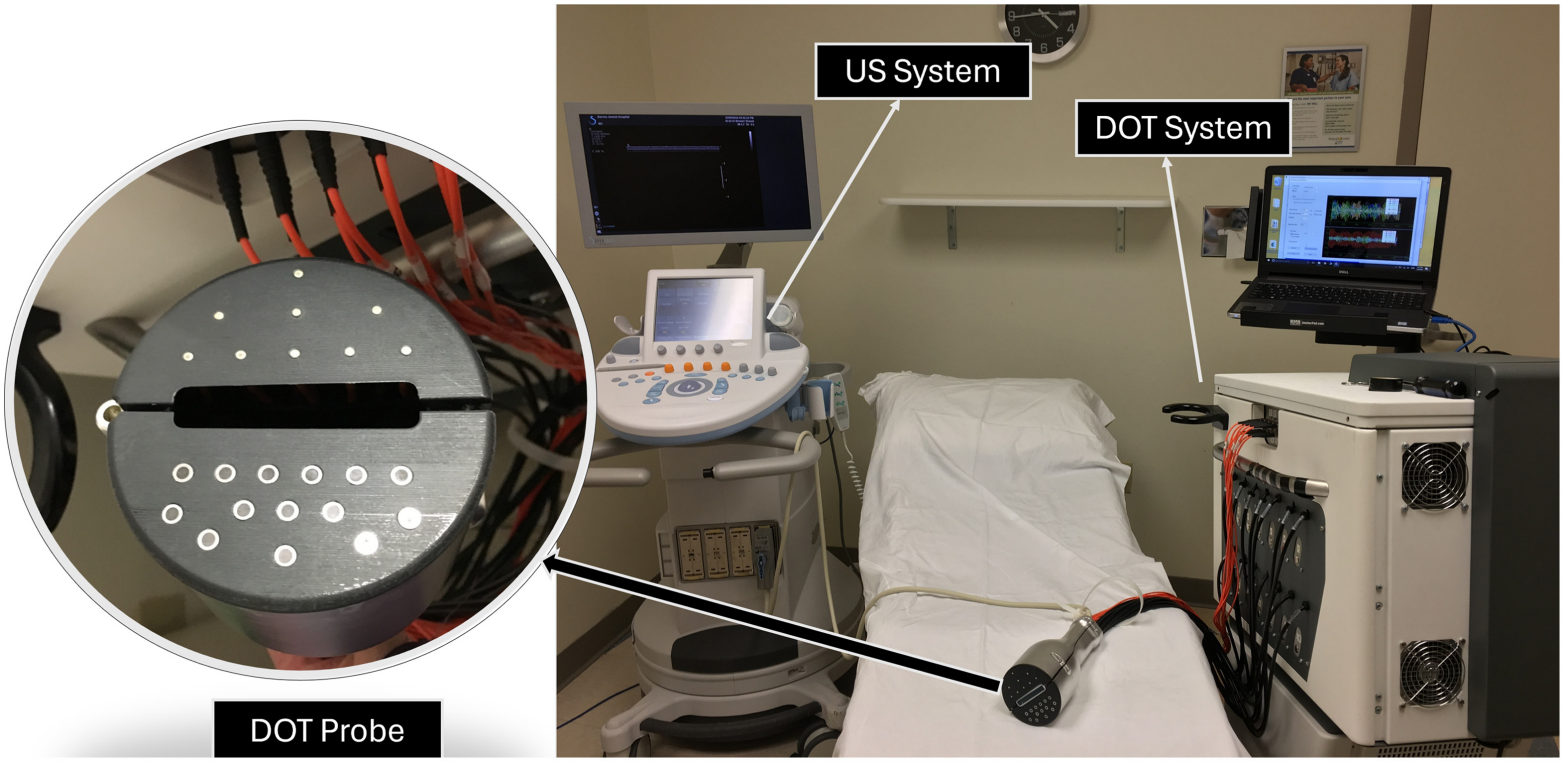
Ultrasound-guided diffused optical tomography system. The handheld probe is shown on the left.

Figure 1 illustrates our ultrasound-guided frequency domain DOT system. It is equipped with a handheld probe designed with 9 optical sources and 14 detectors, with the US transducer positioned in the center. The optical signals from the nine sources, modulated at a frequency of 140 MHz, are transmitted sequentially into the breast tissue. All detectors record signals from each source position simultaneously at the modulated frequency, and then the data acquisition module demodulates the signals to 20 kHz for A/D, resulting in a complete set of 9×14×2 measurements consisting of amplitude and phase. Measurements are taken from both sides of the breast, lesion side as a target and the contralateral side breast as the reference. The perturbation, $U_{\mathrm{SC}}$, is computed using these two sets of measurements, which are then used in the DOT reconstruction process. Although the inverse problem of reconstruction is inherently nonlinear, the Born approximation allows for the transformation of this nonlinear problem into a linear one. The objective function for the inverse reconstruction process, optimized using the regularized conjugate gradient method,^23^^,^^24^ relies heavily on the linear assumption of the Born weight, W, and unknown absorption, $\delta \mu_{a}$

$$\delta {\overset{˜}{\mu }}_{a}=\arg\;\underset{\delta \mu_{a}}{\min}({\left\|U_{\mathrm{SC}}-W\delta \mu_{a}\right\|}^{2}+\frac{\lambda }{2}{\left\|\delta \mu_{a}\right\|}^{2}).$$ (1)

## 3 Methodology

This section presents our proposed reconstruction model, based on the autoencoder and GAN architecture, referred to as DOT-AE-GAN. The model leverages perturbations, $U_{\mathrm{SC}}$, optical properties, and location of the lesion derived from co-registered ultrasound imaging to facilitate DOT reconstruction. We provide a brief overview of computing perturbations from DOT measurements, the calculation of background absorption and scattering coefficients, and the architecture of the DOT-AE-GAN model.

### 3.1 Perturbation

We acquired a set of DOT measurements from the target tissue with a lesion and another set of reference measurements from the contralateral normal breast tissue. We computed $U_{\mathrm{SC}}$ using Eq. (2) derived from these two measurements

$$U_{\mathrm{SC}}=\frac{U_{l}-U_{r}}{U_{r}}=(\frac{A_{l}}{A_{r}}\;\cos(\phi_{l}-\phi_{r})-1)+j\frac{A_{l}}{A_{r}}\;\sin(\phi_{l}-\phi_{r}).$$ (2)

In this context, $U_{l}$ and $U_{r}$ denote the measurements from the lesion side and the reference side, respectively. The $U_{\mathrm{SC}}$ is a matrix of complex numbers, which serves as the input for our reconstruction model.

### 3.2 Background Tissue Absorption and Scattering Coefficients

In the Born approximation–based inverse reconstruction process, we assume that the absorption and scattering coefficients, for the breast tissue around the lesion, are homogeneous. The mean absorption and scattering coefficients are derived from the DOT measurement of the normal reference side. By assuming a semi-infinite medium, we plot the logarithm of the product of the amplitude and the square of the source-detector distance, $\log(A_{r}\rho^{2})$, as well as the phase, $\phi_{r}$, against the source-detector distance, ρ. The slopes, $k_{i}$ and $k_{r}$, of the fitted curves from these plots are subsequently used to determine the background absorption and scattering coefficients, where the two slopes $k_{i}$ and $k_{r}$ represent the imaginary and real parts of the wavenumber k in diffusion theory, respectively.

### 3.3 Model Architecture

**Fig. 2:**
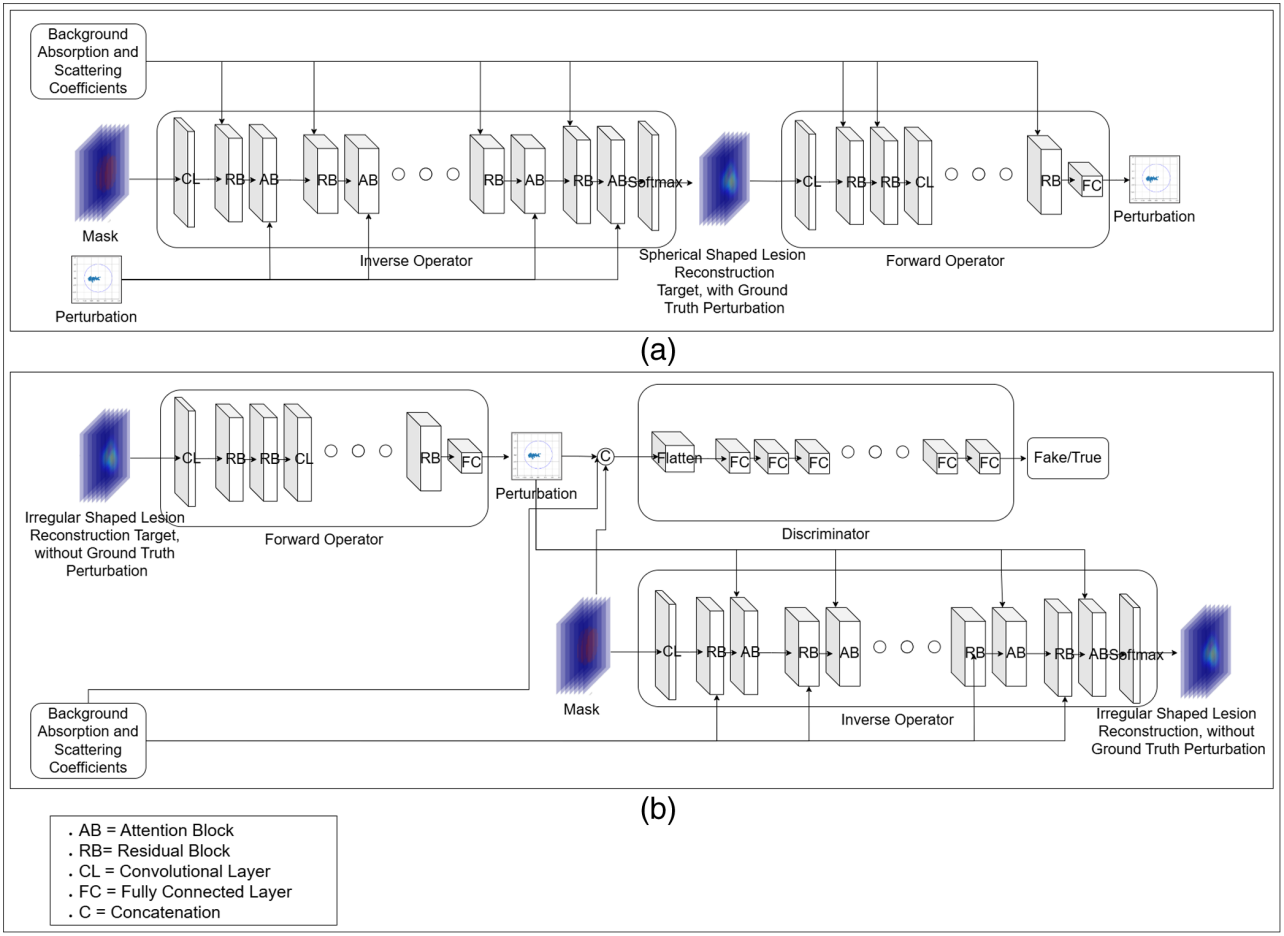
Schematic of the proposed DOT-AE-GAN architecture for DOT reconstruction. (a) Perturbation-to-perturbation (P2P): the lesion mask, perturbation, and background optical properties are input into the inverse operator model to obtain the reconstruction. The forward operator model then processes the reconstruction to predict the original perturbation back. (b) Reconstruction-to-reconstruction (R2R): an irregularly shaped target with unknown perturbation is input into the forward operator model to predict the corresponding perturbation, which is then passed through the inverse operator to recover the reconstruction.

In this section, we detail the architecture of DOT-AE-GAN. A visual representation of the DOT-AE-GAN framework is shown in Fig. 2. The network is composed of three primary components: the forward operator network, the inverse operator network, and the discriminator network. The forward operator simulates the proton transport process in tissue, whereas the inverse operator models the mapping from DOT measurements to reconstructed images. The discriminator network is utilized to guide the training process in an adversarial approach.^22^ We alternated the inverse and forward operators to create two sequences: the perturbation-to-perturbation sequence and the reconstruction-to-reconstruction sequence. The perturbation-to-perturbation sequence is an autoencoder model, following the method in the literature,^14^ and is used to map perturbations to reconstructions and vice versa. Conversely, the reconstruction-to-reconstruction sequence establishes a mapping between reconstructions and perturbations and vice versa. This sequence is conceptualized as a generative adversarial network, wherein the discriminator is utilized to train the generator model, which, in this context, corresponds to the forward operator.

#### 3.3.1 Inverse operator network

We designed the inverse operator network to model the inverse process of reconstruction. We provide the inverse operator network with three inputs: spherical mask, perturbation, and background tissue absorption and scattering coefficients, resulting in the reconstruction of the DOT image as the output, R^. For the inverse operator illustrated in Fig. 2(a) (left) and Fig. 2(b) (lower right), we implemented a U-Net architecture augmented with transformer layers, following the approach described in the publication^25^

R^=INV(M,Usc,B). (3)

**Fig. 3:**
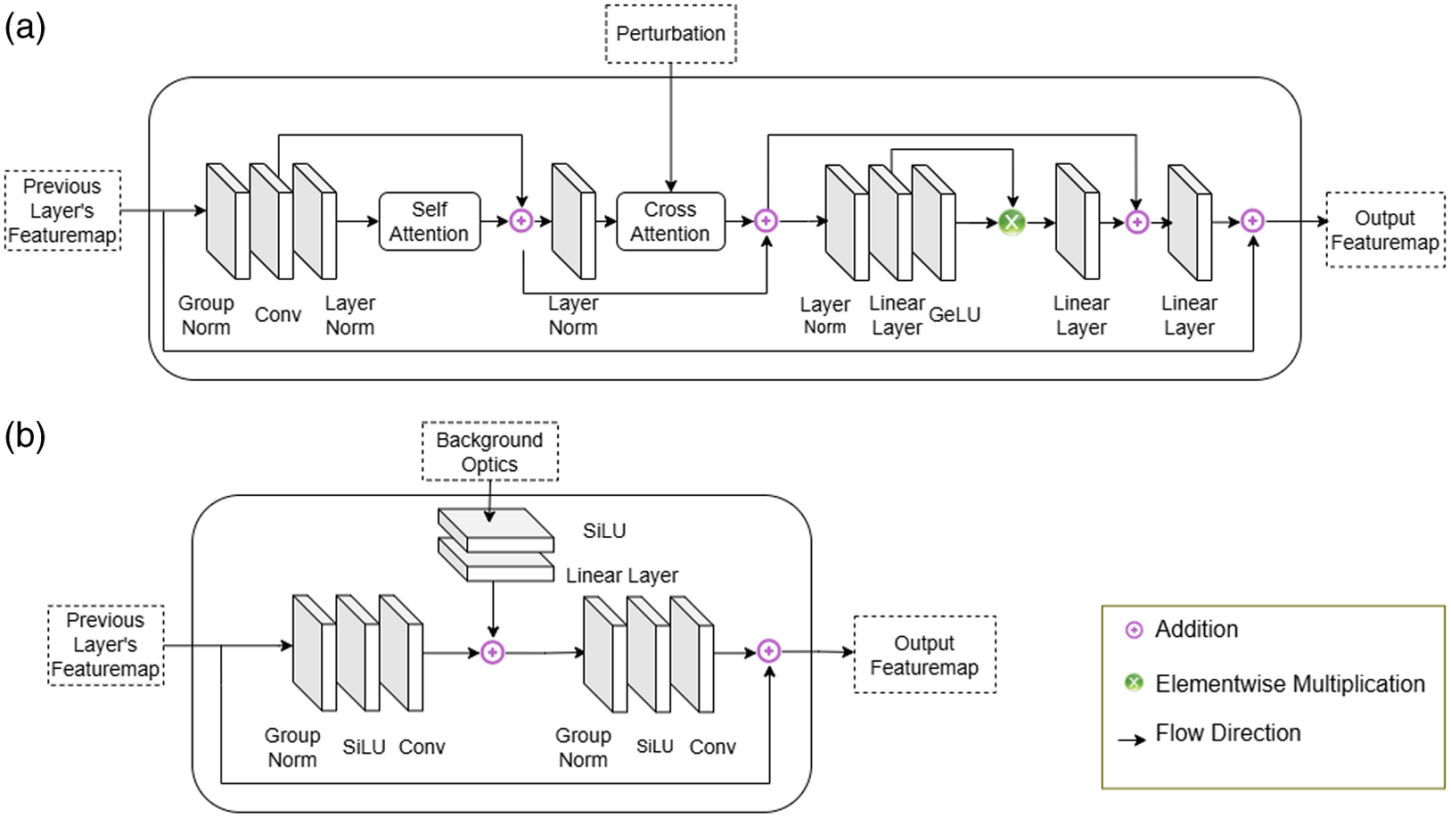
Building blocks of neural networks. (a) Attention block (AB) containing self-attention and cross-attention. (b) Residual block (RB) containing residual connections with absorption coefficient and scattering coefficient of background tissue.

Here, Eq. (3) defines the inverse operator with three inputs: M, a spherical mask; $U_{\mathrm{sc}}$, the perturbation; and B, the background tissue absorption and scattering coefficient. To estimate the lesion mask, we utilized the co-registered ultrasound images acquired concurrently with the DOT measurements. From these US images, we assessed the depth of the lesion from the surface and approximated its radius in spatial and depth dimensions. Based on these parameters, we generated a spherical mask to guide the neural network in reconstruction. The mask size was intentionally varied between two to three times the actual lesion dimensions to account for potential larger boundary or size measured in US images. The inverse operator is built with alternating attention block [Fig. 3(a)] and residual block [Fig. 3(b)], where the attention block is driven by the perturbation data and the residual block is driven by the background optical properties. The layout of the attention block and residual block is depicted in Fig. 3. In the attention block, we have used self-attention and cross-attention layers^26^ defined by Eq. (4)

$$\text{Attention}(Q,K,V)=\text{softmax}(\frac{QK^{⊤}}{\sqrt{d_{k}}})V,$$ (4)

where, in the case of the cross-attention layer, the query matrix Q is derived from the feature map of the previous layer, whereas the key K and value V matrices are obtained from the perturbation input $U_{\mathrm{SC}}$. In contrast, for the self-attention layer, all three matrices—Q, K, and V—are computed from the previous layer’s feature map. Here, the feature map refers to the multi-dimensional output tensor generated by a specific network layer, typically encoding spatial and semantic features. The previous layer denotes the layer immediately preceding the current one in the architecture, from which the featuremap is obtained. We introduced cross-attention layers to handle perturbation inputs with greater precision, complemented by self-attention layers to ensure that critical parameters from the previous layers are adequately emphasized. The inclusion of cross-attention enables more precise handling of perturbation inputs, whereas self-attention helps retain and emphasize critical features propagated through the network. This design facilitates a deeper and more context-aware encoding of measurement perturbations.

#### 3.3.2 Forward operator network

The forward operator network is designed to model the photon propagation process through tissue. The forward operator is a feed-forward network, incorporating residual blocks, Fig. 3(b), and convolutional layers^27^ in its backbone. The forward operator is depicted in Fig. 2(a) (right) and Fig. 2(b) (upper-left)

U^sc=FWD(R,B). (5)

The reconstructed absorption map contains only the absorption coefficient values of the lesion and the surrounding tissue. However, to effectively model the forward process of photon propagation, we incorporated the background absorption and scattering coefficients as needed parameters in our forward model to derive the perturbation. Equation (5) represents the forward operator, where R represents the reconstructed image and B contains the background absorption and scattering coefficients.

#### 3.3.3 Discriminator network

The discriminator network is a feed-forward architecture comprising a series of basic linear perceptron layers.^28^ This network facilitates the adversarial training of our DOT-AE-GAN model. Its architecture is depicted in Fig. 1(b) (upper-right). The network takes the perturbation, U^sc, generated by the forward operator network, along with the maximum absorption coefficients, max(R), obtained from the reconstructed DOT as input. It also includes the background absorption and scattering coefficients, B, and the spherical mask of the lesion, M. Based on these inputs, it assesses the quality of the predicted perturbation by comparing it to finite element method (FEM)- and Monte Carlo (MC)-simulated perturbations. The discriminator is mathematically expressed by Eq. (6), which provides a comprehensive representation of its underlying principles and functionality within the broader framework of the model

D^=D(M,B,U^sc,max(R)). (6)

### 3.4 Perturbation-to-Perturbation Sequence

We sequentially placed the inverse operator followed by the forward operator to obtain perturbations for reconstruction and vice versa. For the inverse operator, the inputs include a spherical mask of the lesion location, the perturbation data, and the lesion’s background tissue absorption and scattering coefficient values. The expected output from the inverse operator is the reconstructed absorption coefficient image. In the forward operator, we used the reconstructed image along with the background absorption and scattering coefficients as inputs. The forward operator’s output is expected to return the same perturbation that led to the reconstruction. The perturbation-to-perturbation sequence can be mathematically expressed as a function shown in Eq. (7)

U^sc=FWD(INV(M,USC,B),B). (7)

### 3.5 Reconstruction-to-Reconstruction Sequence

We used FEM-simulated data and Monte Carlo–simulated data to train our model. However, generating a wide range of diverse irregular shapes that could potentially represent breast lesions is challenging for such types of simulations. To enhance the robustness of our algorithm for reconstructing irregularly shaped lesions, we generated a variety of reconstructed targets with diverse irregular shapes or geometries. However, the corresponding perturbations of these reconstructions were unknown. To address this, we adopted a GAN approach to train the network using these reconstruction targets with unknown perturbations. We sequenced the forward operator and the inverse operator so that we can feed those irregular targets to obtain perturbations and vice versa. We generated a variety of reconstructed images with Gaussian mixture density and expected the forward operator to provide perturbations corresponding to these reconstructions. These perturbations were then fed into the inverse operator to predict back the reconstructed images. The mathematical function representing the reconstruction-to-reconstruction sequence is provided in Eq. (8)

R^=INV(FWD(R,B),M,B). (8)

To ensure the forward operator produces valid perturbations, we employed a discriminator model. The discriminator guides the forward operator to generate realistic perturbations for a given reconstruction, based on the background scattering and absorption coefficients. We trained the discriminator by assigning the perturbations generated through our simulation methods as real or true perturbations while assigning those perturbations produced by the forward operator, using the generated unrealistic lesions as fake. By doing so, we effectively train the sequence and force the forward operator to produce perturbations within the true distribution for the irregularly shaped lesions.

## 4 Training and Loss Functions

Unlike the ML-PC^14^ model, which explicitly integrates a physical constraint via a Born-weighted loss, the DOT-AE-GAN framework adopts a fundamentally different approach. In our design, the optical absorption and scattering coefficients of the background tissue are fed into the model based on the Born approximation, thereby inherently embedding a Born-based constraint into the model. We have found that further addition of Born-weighted loss during training was not needed. We trained the model by alternating between two training sequences: perturbation-to-perturbation (P2P) and reconstruction-to-reconstruction (R2R). Each iteration of training involved two sequential steps-first optimizing the P2P sequence, followed by the R2R sequence. This alternating scheme was repeated within every training step for a batch of data in each epoch.

In the P2P sequence, both the forward model (FWD) and the inverse model (INV) were trained using simulated data, for which ground truth perturbations and corresponding reconstructions were available. This step was supervised and straightforward, with the loss defined in Eq. (9):

LP2P(INV,FWD)=1N∑i=1N[|Ri−R^i|+|Ri−R^iRmaxi|+|USCi−U^sci|]. (9)

In the R2R sequence, the model was trained on reconstructions with irregular lesion shapes, for which no ground truth perturbations exist. To ensure the generated perturbations remained physiologically plausible, a discriminator (D) was introduced to form an adversarial learning setup. During this phase, both FWD and INV were updated to minimize the total loss, whereas D was updated to maximize the adversarial component. The combined objective function for training this sequence is expressed in Eq. (12)

$$L_{\mathrm{GAN}}(\mathrm{FWD},D)=E_{p}[\log\;D(M,B,U_{\mathrm{SC}},\max(R))]+E_{R,B}[\log(1-D(M,B,\mathrm{FWD}(R,B),\max(R)))],$$ (10)

LR(FWD,INV)=1N∑i=1N[|Ri−R^i|+|Ri−R^iRmax,i|], (11)

$$L_{R2R}(\mathrm{FWD},\mathrm{INV},D)=\underset{\mathrm{FWD},\mathrm{INV}}{\arg\;\min}\underset{D}{\max}(L_{R}+\lambda L_{\mathrm{GAN}}).$$ (12)

Here, λ=0.001 balances the reconstruction and adversarial losses. Importantly, only one set of optimizers was used for the shared FWD and INV networks across both sequences. However, the discriminator D was only updated during the R2R training step and remained frozen during P2P training. No additional model weights were fixed or frozen beyond this. This design ensured stable and efficient convergence of the entire model across both supervised and adversarial domains.

## 5 Experimental Results

We conducted a series of experiments using simulation, phantom, and clinical data to validate the effectiveness of our method. All reconstructions were performed using data obtained at 808-nm wavelength. To assess the model’s performance in enhancing lateral resolution, we have used contour level as a metric. Equation (13) defines the contour for the absorption map. The diameter, D, of the contour can be calculated based on the spatial coordinates $\left(x_{i},y_{i}\right)$ of the points lying on the contour using Eq. (14)

C=0.5·max(f(x,y)), (13)

$$D=\max(\sqrt{(x_{i}-x_{j}{)}^{2}+(y_{i}-y_{j}{)}^{2}}).$$ (14)

Our results demonstrate that DOT-AE-GAN outperforms and exhibits robustness compared with US-guided regularized Born-Conjugate Gradient (Born-CGD)^24^ and ML-PC.^14^ A summary of the model’s performance across different data types is provided below.

### 5.1 Simulation Results

We trained our model on simulated breast lesions of varying sizes and absorption coefficients. The lesions ranged from 0.45 to 1.2 cm in radius, and their absorption coefficients varied between 0.10 and $0.30\;{\mathrm{cm}}^{-1}$. To simulate different types of breast tissue and create challenging scenarios for the reconstruction algorithm, we also varied the background absorption and scattering coefficients in simulations with an absorption coefficient between 0.02 and $0.09\;{\mathrm{cm}}^{-1}$, and a scattering coefficient between 4 and $9\;{\mathrm{cm}}^{-1}$. Table 1 shows the number of different types of data used to train our model.

**Table 1:** Training and testing data.

Data type	Data source	Data set number
Training	FEM and MC simulation (known ground truth perturbation)	21,177
Training	Irregularly shaped lesion (unknown ground truth perturbation)	255,988
Testing	FEM and MC simulation (known ground truth perturbation)	2353

**Fig. 4:**
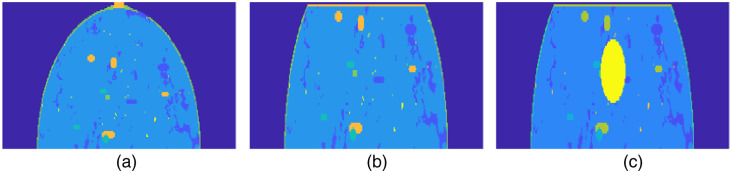
Preprocessing of the breast phantom prior to Monte Carlo simulation. (a) Noise introduction to the phantom. (b) Compression to form a flat surface. (c) Placement of lesion.

We utilized three distinct datasets at various stages of training and testing the DOT-AE-GAN. For simulation, the Monte Carlo simulation method was employed, leveraging a digital breast phantom developed by Park et al.^29^ Figure 4 presents a sagittal view of the phantom. To simulate our imaging process, we compressed the digital phantom to create a flat surface at the top, where the handheld transducer probe is positioned for imaging. Subsequently, spherical lesions with varying absorption coefficients were introduced at different depths to replicate breast lesions.

**Fig. 5:**
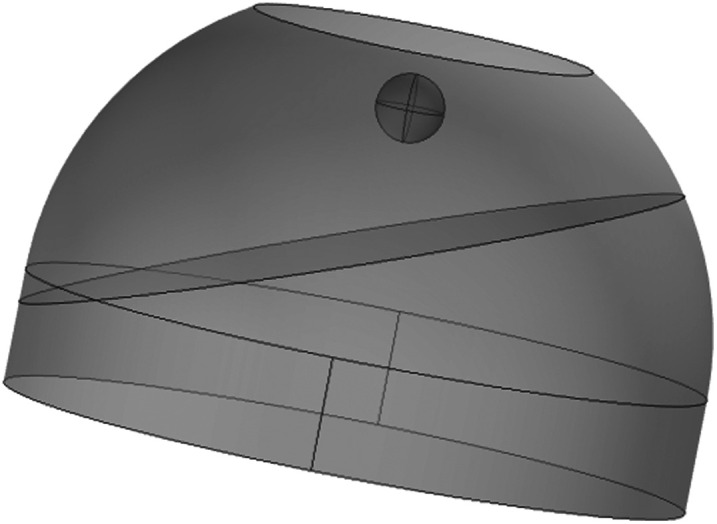
Digital phantom for finite element method simulation.

Another digital phantom, designed in our lab, was used to generate simulation data. Figure 5 illustrates this phantom, where a plane with different optical properties from breast tissue was induced to represent the chest wall, and a spherical ball placed at different depths represents the target breast lesion. This simulation was conducted using the COMSOL software, with the finite element method governed by the diffusion equation.

We simulated 23,530 samples using these 2 types of simulation setups. Where around 2800 samples were generated using MC simulation and the rest with the COMSOL FEM setup. In both simulation setups, spherical lesions were placed at depths ranging from 0.5 to 3 cm. The radius of the lesions varied between 0.5 and 1.2 cm. The absorption coefficients of the spherical lesions ranged between 0.1 and $0.3\;{\mathrm{cm}}^{-1}$. We take one set of measurements prior to the introduction of the lesion and another set of measurements after breast legion introduction. Later, these two measurements are used to calculate the perturbation.

To make our model robust for irregularly shaped lesion reconstruction, we generated irregularly shaped targets. The process begins by defining the depth and approximate dimensions of the lesion. Next, one to three random islands are generated within these specified dimensions to create the foundational structure. These islands are then smoothed using Gaussian blurring to ensure a more natural appearance. Following this, the pixel values are normalized to maintain consistency and prepare the data for further processing. Finally, the normalized values are multiplied by the maximum absorption level to complete the transformation.

**Fig. 6:**
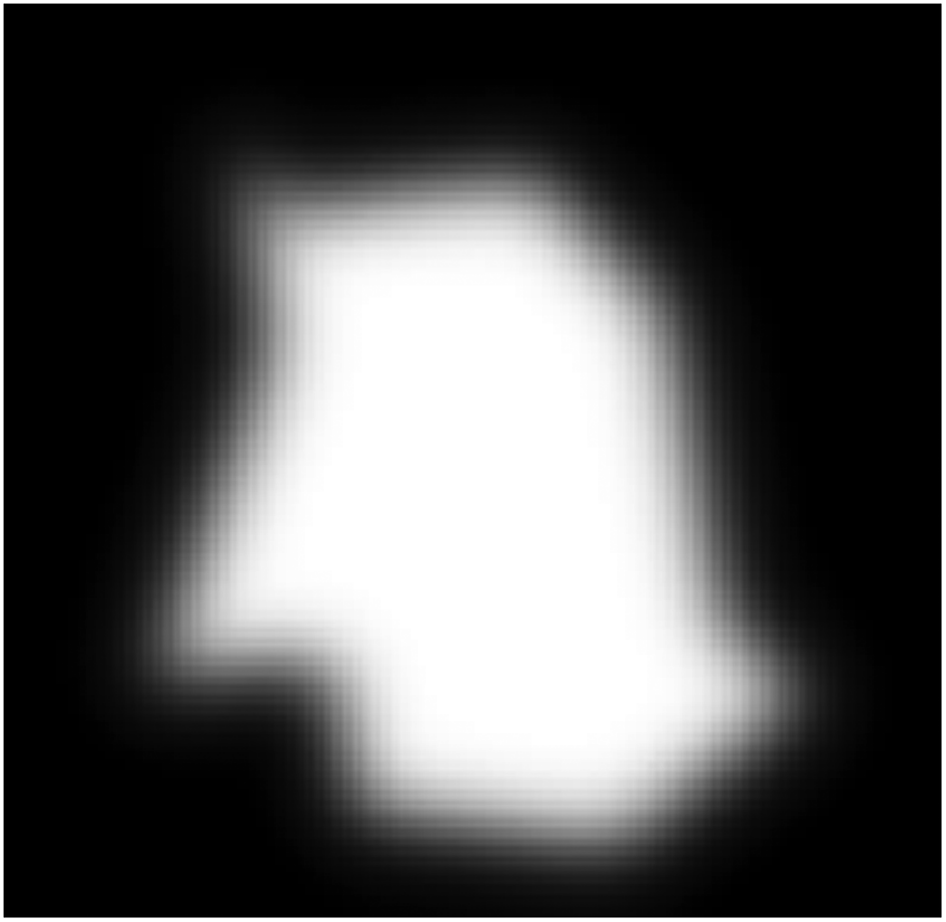
Example of a two-dimensional slice extracted from a 3D lesion target, showing an irregularly shaped lesion.

Figure 6 shows an example transverse slice extracted from one of the three-dimensional (3D) irregular lesion targets used for training. As ground truth perturbations for such irregular shapes were unavailable, we leveraged a GAN-based generative training approach to learn from these reconstruction targets without explicit perturbation labels.

#### 5.1.1 Maximum absorption coefficient

**Fig. 7:**
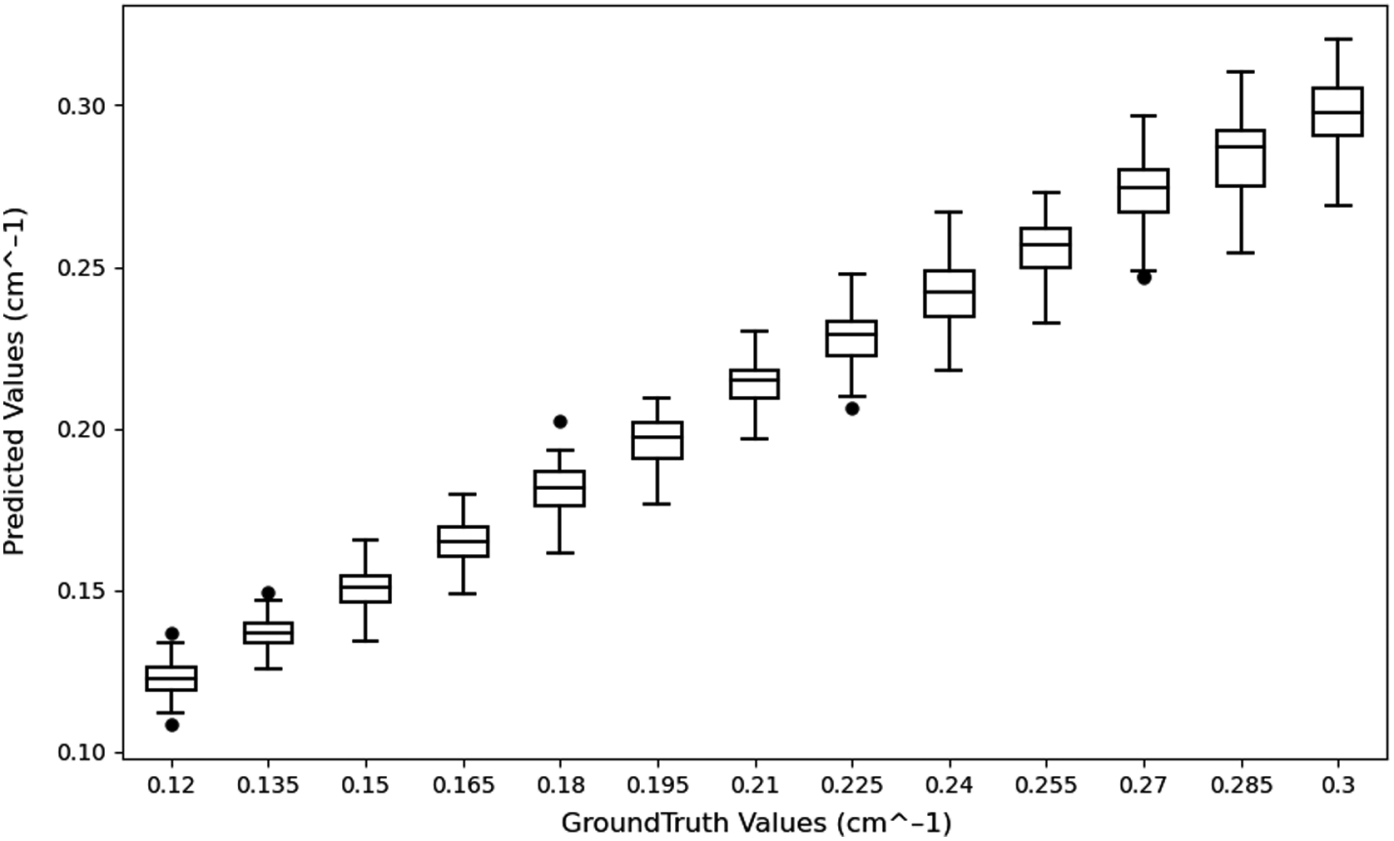
Reconstructed maximum absorption coefficient (${\mathrm{cm}}^{-1}$) for simulated test data.

We have used the maximum absorption coefficient calculated from the absorption map to assess different reconstruction algorithms. This measure has been used by us and others to evaluate the reconstruction accuracy. In our evaluation of 2353 test samples, we observed that the maximum absorption coefficients obtained were nearly equivalent to the ground truth values. The accompanying box plot, Fig. 7, illustrates the comparison between the reconstructed maximum absorption coefficients and the ground truth values. The ground truth absorption coefficients and predicted absorption coefficients almost performed linearly with an $R^{2}$ value of 0.976.

#### 5.1.2 Lesion’s lateral dimension

**Fig. 8:**
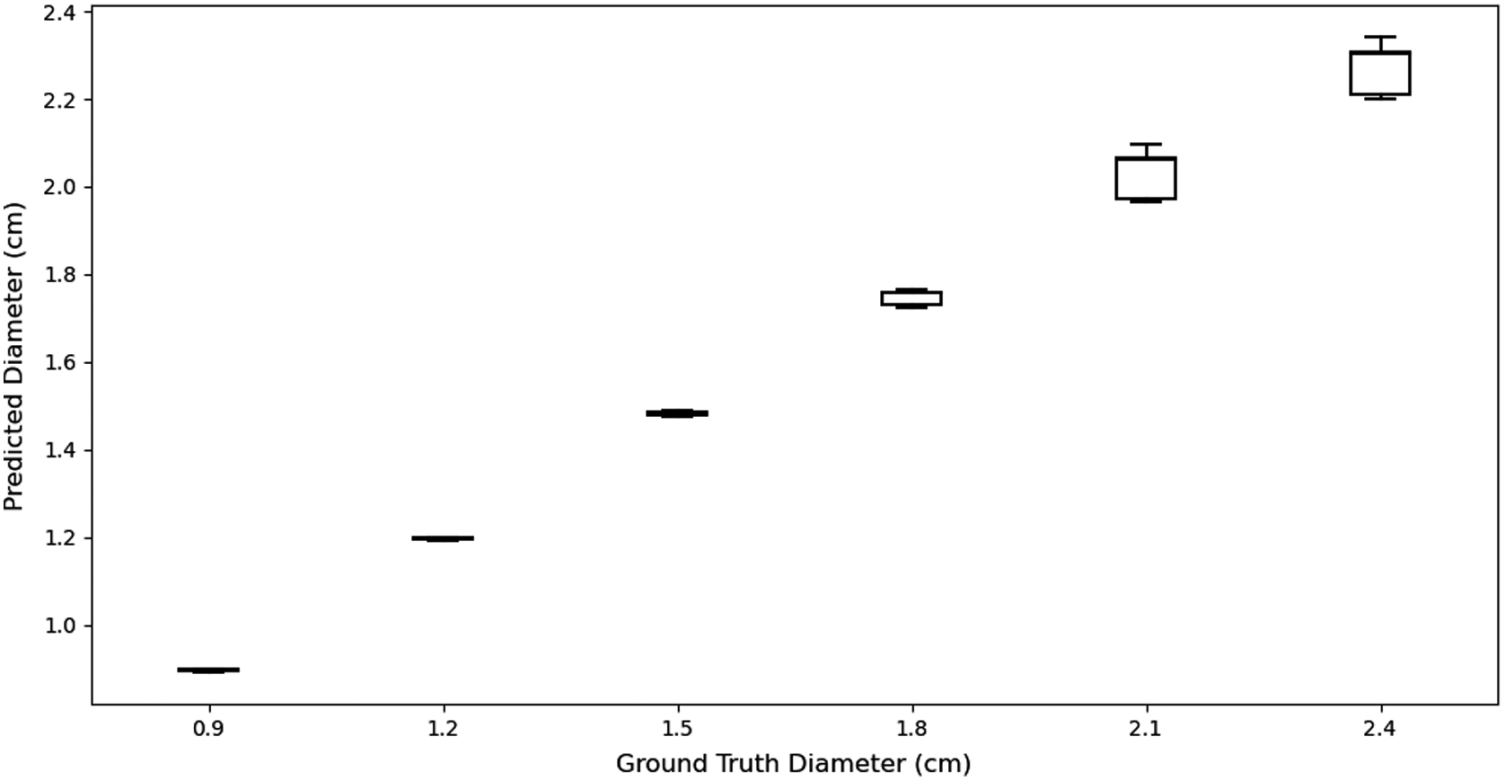
Reconstructed lesion diameter at 50% contour (cm) for simulated test data.

DOT is a low-resolution functional imaging modality, and the classical reconstruction algorithms^23^^,^^24^ find it very difficult in terms of estimating lateral dimensions of the lesion. One key feature of our DOT reconstruction algorithm is to enhance the lateral resolution. Figure 8 shows the distribution of 50% contour level diameter of the reconstructed lesion against the ground truth diameter. We observed that the variance in the reconstructed lesion diameter increases with the lesion’s size. This is attributed to the measurement of the diameter being based on the contour level. Nevertheless, we found the performance of our model in terms of lateral resolution to be robust and linear with an $R^{2}$ value of 0.979.

### 5.2 Phantom Results

**Fig. 9:**
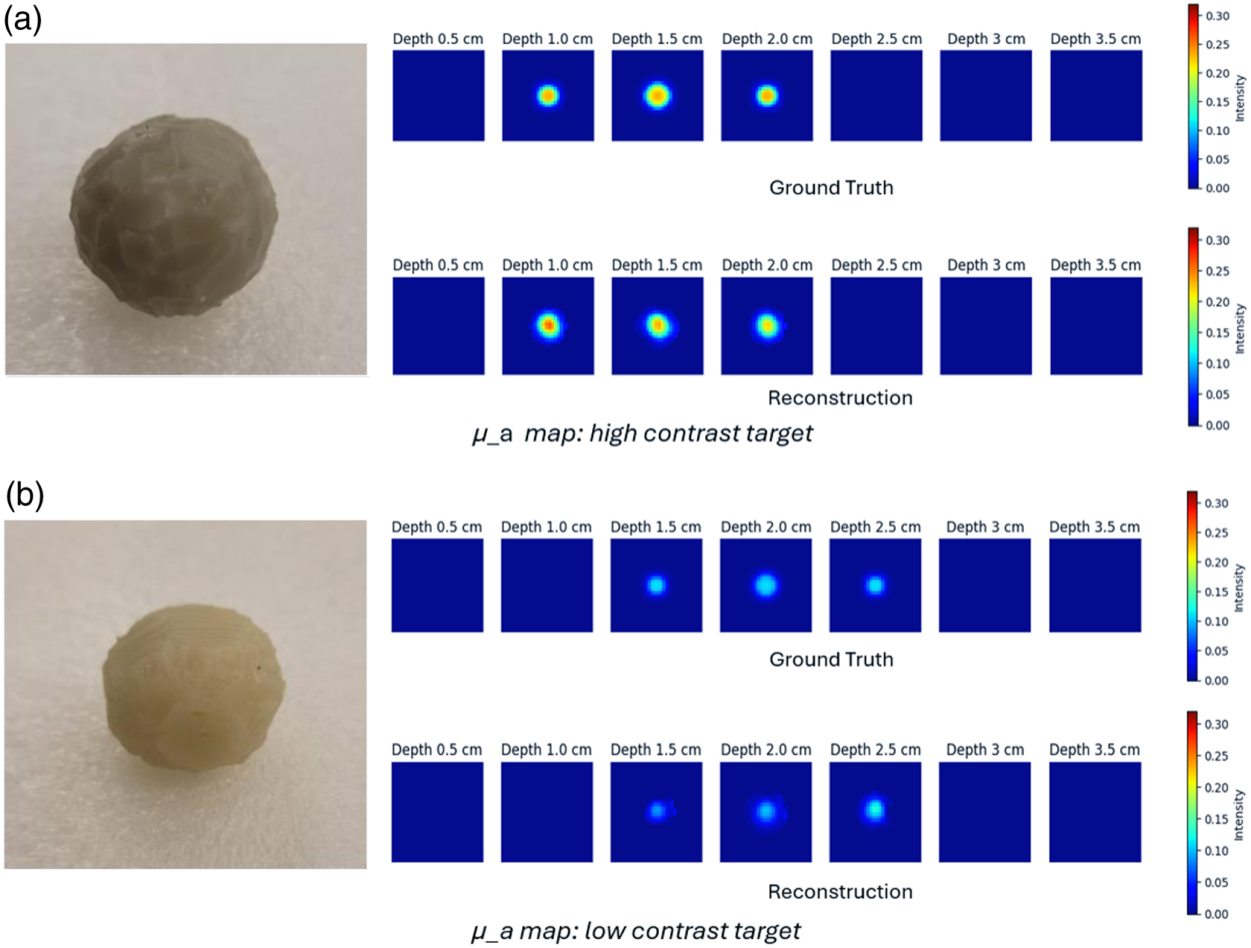
(a) Reconstruction on high absorption target of $0.23\;{\mathrm{cm}}^{-1}$. μ_a map: high-contrast target. (b) Reconstruction on low absorption target of $0.11\;{\mathrm{cm}}^{-1}$. μ_a map: low-contrast target.

We used a modest collection of 90 samples as phantom data to evaluate our model’s performance in a controlled setting, including both spherical and irregular, nonspherical inclusions. We utilized 50% of the data to fine-tune the model, which enhances its robustness for patient data. The remaining 50% was used to evaluate the model’s performance. Sample phantoms along with their corresponding reconstructed images are shown in Fig. 9, where each of them has different absorption coefficients of $0.23\;{\mathrm{cm}}^{-1}$ (black) and $0.11\;{\mathrm{cm}}^{-1}$ (white).

#### 5.2.1 Maximum absorption coefficient

**Fig. 10:**
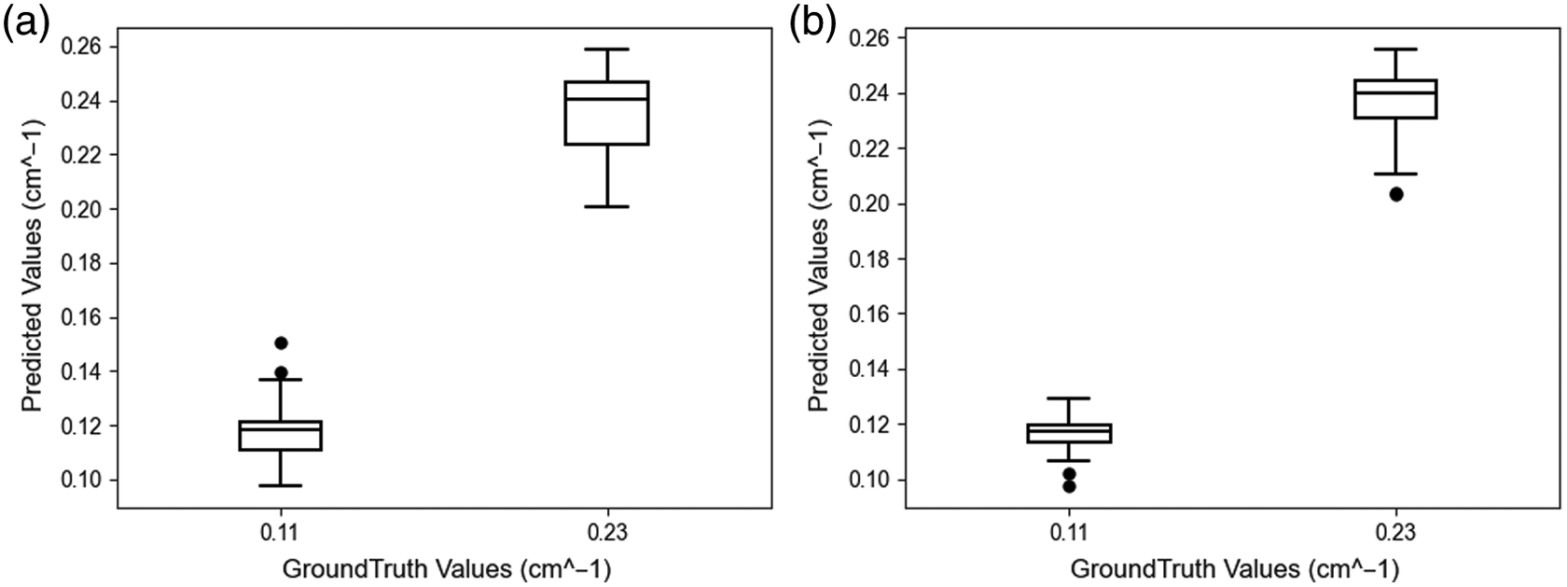
Maximum absorption coefficient from DOT reconstruction on phantoms against the ground truth maximum absorption coefficient. (a) ML-PC. (b) DOT-AE-GAN.

Figure 10 presents box plots comparing the reconstructed maximum absorption coefficients of ML-PC^14^ and DOT-AE-GAN against the ground truth maximum absorption coefficients. These visualizations provide a depiction of the distribution and variability in the reconstructed values for both models. The results indicate that both ML-PC^14^ and DOT-AE-GAN provide similar reconstructed target absorption; however, DOT-AE-GAN exhibits lower variance (Table 2), suggesting its enhanced consistency in capturing the underlying absorption characteristics.

**Table 2:** Quantitative comparison of absorption coefficient (${\mathrm{cm}}^{-1}$) with phantom data.

True value	ML-PC		DOT-AE-GAN	
	Mean	95% CI	Mean	95% CI
0.11	0.117 ± 0.009	[0.114, 0.121]	0.116 ± 0.006	[0.114, 0.118]
0.23	0.235 ± 0.016	[0.230, 0.240]	0.236 ± 0.012	[0.232, 0.239]

#### 5.2.2 Lesion’s lateral dimension

**Fig. 11:**
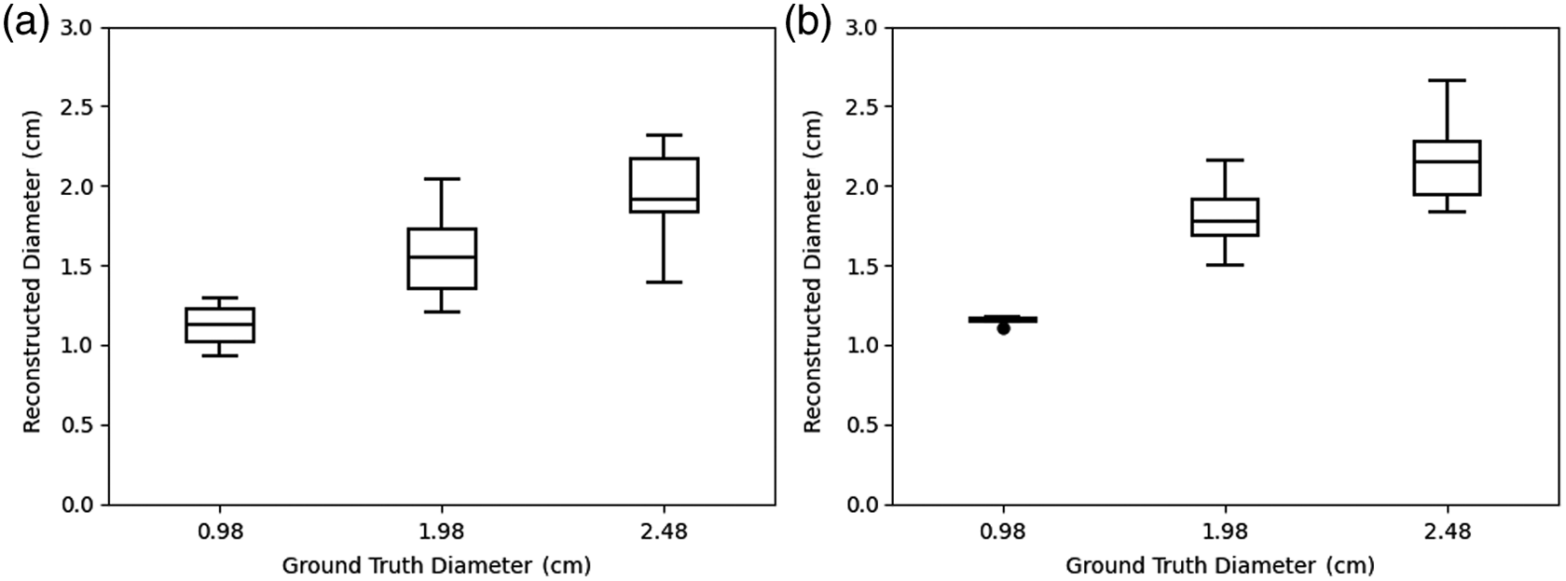
Reconstructed lesion diameter at 50% contour level against the ground truth diameter. (a) ML-PC. (b) DOT-AE-GAN.

For DOT reconstruction, we provided lesion masks obtained from co-registered ultrasound. Figure 11 depicts the reconstructed lesion diameter for both ML-PC^14^ and DOT-AE-GAN, at the 50% contour level relative to the true diameter. Table 3 complements this visualization by providing a detailed quantitative analysis. The results indicate that DOT-AE-GAN performs significantly better than ML-PC,^14^ exhibiting lower variance and improved accuracy in reconstructing target size.

**Table 3:** Quantitative comparison of reconstructed lesion diameter (cm) with phantom data.

True diameter	ML-PC		DOT-AE-GAN	
	Mean	95% CI	Mean	95% CI
0.98	1.120 ± 0.137	[0.992, 1.247]	1.149 ± 0.022	[1.128, 1.169]
1.98	1.554 ± 0.231	[1.468, 1.641]	1.792 ± 0.182	[1.723, 1.859]
2.48	1.933 ± 0.290	[1.804, 2.061]	2.148 ± 0.205	[2.062, 2.235]

### 5.3 Clinical Data Results

**Fig. 12:**
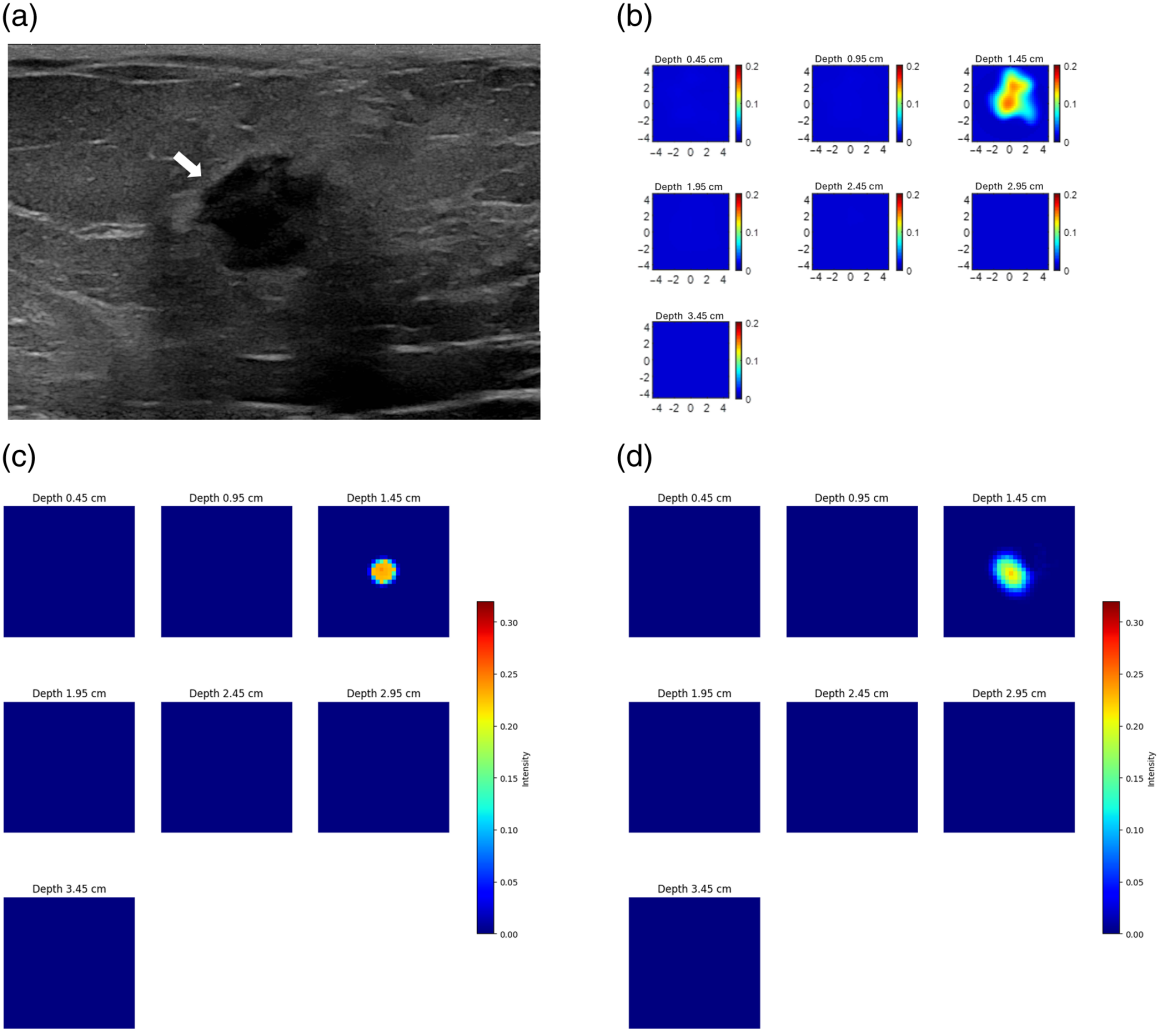
78-year-old patient with an invasive ductal carcinoma. (a) US image. Co-registered ultrasound showed an irregular lesion shape with the largest diameter of ∼1.6  cm. (b) DOT-reconstructed absorption coefficient map using the regularized CGD-based reconstruction. CGD, max $\mu_{a}$: $0.151\;{\mathrm{cm}}^{-1}$. (c) ML-PC-based reconstruction, max $\mu_{a}$: $0.235\;{\mathrm{cm}}^{-1}$. (d) DOT-AE-GAN-based reconstruction, max $\mu_{a}$: $0.223\;{\mathrm{cm}}^{-1}$. Note that seven slices were reconstructed in each DOT map [panels (b)–(d)], and each slice represents depth from the breast surface as marked with x-y dimensions of 9×9  cm. As shown in panel (b), the reconstructed lesion spreads into a larger spatial dimension than that ultrasound-measured dimension. Both ML-PC and DOT-AE-GAN reconstructed localized absorption maps corresponding to the ultrasound lesion location. DOT-AE-GAN recovered the lesion more smoothly due to the GAN-assisted training.

**Fig. 13:**
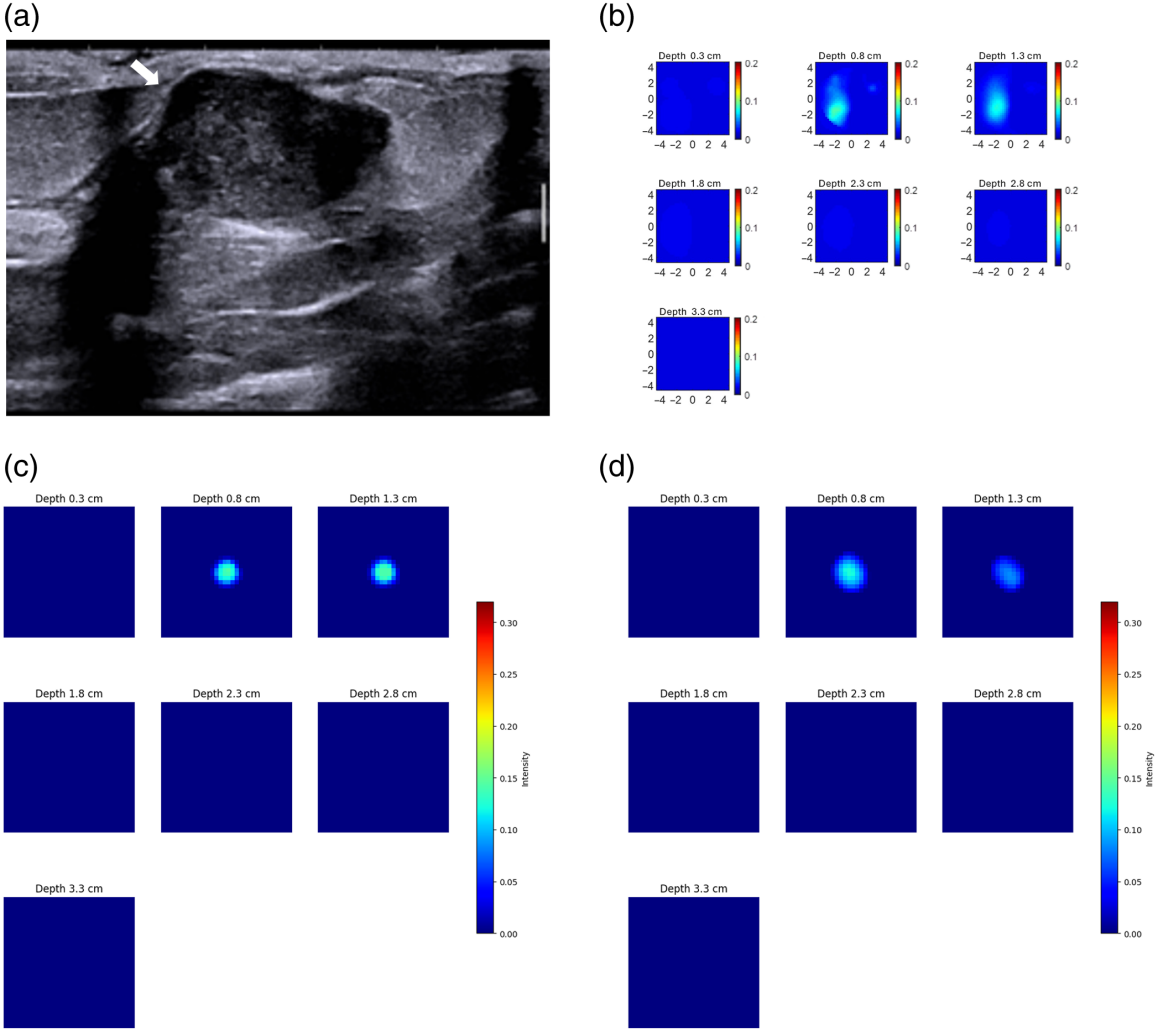
37-year-old patient with a benign fibroadenoma. (a) US image. Co-registered ultrasound image. (b) CGD-based reconstruction, max $\mu_{a}$: $0.093\;{\mathrm{cm}}^{-1}$. (c) ML-PC-based reconstruction, max $\mu_{a}$: $0.131\;{\mathrm{cm}}^{-1}$. (d) DOT-AE-GAN-based reconstruction, max $\mu_{a}$: $0.124\;{\mathrm{cm}}^{-1}$. Again, both ML-PC and DOT-AE-GAN reconstructed localized absorption maps corresponding to the ultrasound lesion location. DOT-AE-GAN recovered the lesion more smoothly due to the GAN-assisted training.

**Fig. 14:**
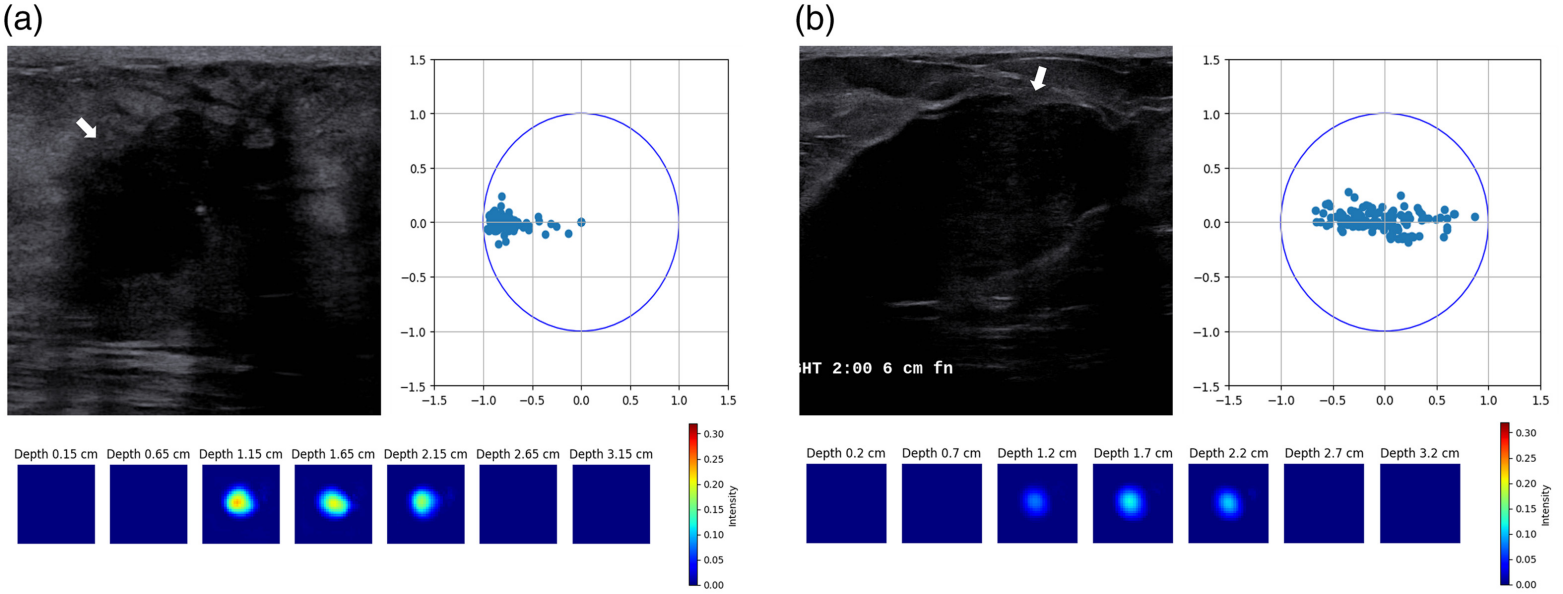
Examples from patient data. (a) Malignant case of US image (top left), corresponding perturbation (top right), and the bottom of reconstructed DOT spatial absorption maps at different depths as marked. The patient is a 67-year-old woman with a high-grade invasive lobular carcinoma. The maximum reconstructed absorption coefficient is $0.23\;{\mathrm{cm}}^{-1}$. (b) Benign case of US image (top left), perturbation (top right), and DOT reconstructed spatial absorption maps at different depths. The maximum reconstructed absorption coefficient is $0.12\;{\mathrm{cm}}^{-1}$. The patient is an 18-year-old woman with a benign fibroadenoma. (a) max $\mu_{a}$: $0.229\;{\mathrm{cm}}^{-1}$. (b) max $\mu_{a}$: $0.120\;{\mathrm{cm}}^{-1}$.

To evaluate our algorithm with clinical data, we utilized a dataset of 40 patients. This dataset included 20 patients with malignant lesions and the remaining patients with benign lesions. The data were obtained using our ultrasound-guided DOT imaging system shown in Fig. 1. Figures 12 and 13 present reconstructions from patient data using CGD, ML-PC,^14^ and DOT-AE-GAN, depicting one malignant lesion and one benign lesion. Figure 14 presents two additional example reconstructions: one of a malignant lesion and one of a benign lesion, along with corresponding measurement perturbation plots and co-registered ultrasound images.

#### 5.3.1 Maximum absorption coefficient

**Fig. 15:**
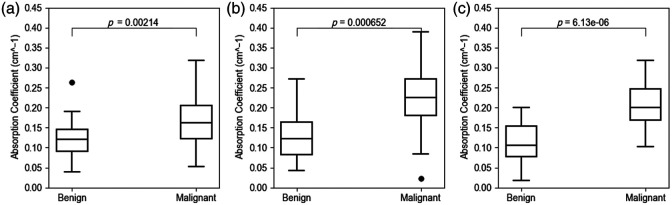
Maximum absorption coefficients for benign and malignant cases. (a) Regularized CGD reconstruction. (b) ML-PC. (c) DOT-AE-GAN.

We compared the maximum absorption coefficients of reconstructed images between benign and malignant groups. In addition, we used Born-regularized CGD^24^ and ML-PC^14^ methods for image reconstruction to benchmark DOT-AE-GAN’s performance. The comparison is illustrated in the box plot shown in Fig. 15. DOT-AE-GAN demonstrates a high-contrast difference in the distribution of the reconstructed maximum absorption coefficients compared with the regularized CGD method for both benign and malignant lesions. Again, DOT-AE-GAN shows lower variance for both benign and malignant lesions than the ML-PC^4^ model. Table 4 shows a quantitative comparison among the regularized CGD method, ML-PC,^14^ and DOT-AE-GAN. This highlights our model’s effectiveness in distinguishing the benign and malignant categories.

**Table 4:** Comparison of the mean absorption coefficients (${\mathrm{cm}}^{-1}$) and standard deviation for benign and malignant groups.

Group	CGD		ML-PC^14^		DOT-AE-GAN	
	Mean	95% CI	Mean	95% CI	Mean	95% CI
Benign	0.120 ± 0.051	[0.099, 0.140]	0.132 ± 0.062	[0.102, 0.161]	0.115 ± 0.051	[0.091, 0.138]
Malignant	0.176 ± 0.069	[0.145, 0.207]	0.222 ± 0.085	[0.181, 0.262]	0.204 ± 0.053	[0.179, 0.229]

#### 5.3.2 Lesion’s lateral dimension

**Fig. 16:**
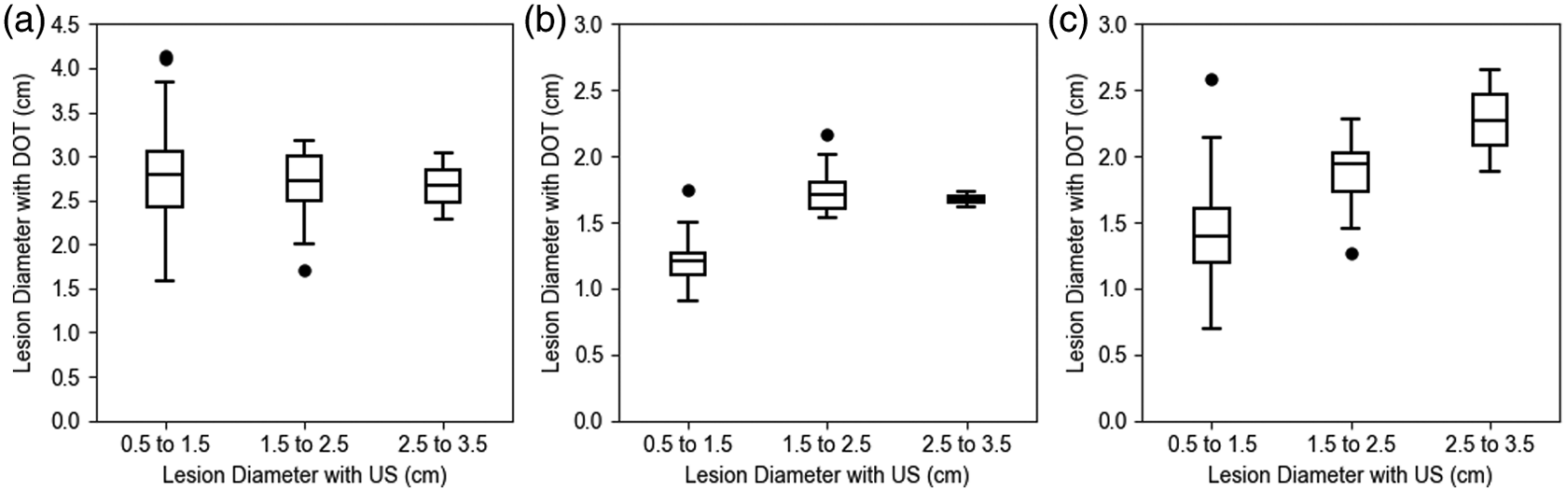
Comparison of lateral diameters of lesions obtained from patient ultrasound images and the co-registered DOT-reconstructed images. (a) CGD. (b) ML-PC. (c) DOT-AE-GAN.

In DOT reconstruction, the regularized CGD^24^ method is used by many research groups. However, due to the intense light scattering in tissue, the regularized CGD method is optimized for improving the convergence of reconstruction when the signal-to-noise ratio of DOT measurement is low. The method is not optimal in recovering lesion dimensions, with notable discrepancies between the reconstructed DOT images and the actual lesion sizes. In contrast, the proposed DOT-AE-GAN model demonstrates superior performance in preserving lesion geometry, providing a more accurate estimation of lesion sizes in reconstructed images. We evaluated its performance using clinical data. As both ultrasound images and DOT measurements are co-registered, we manually measured the largest diameter from the ultrasound B-scan image of each lesion and then compared it with the largest diameter of 50% contour level of the DOT absorption map. The results for three lesion sizes are illustrated in Fig. 16, with corresponding quantitative comparisons provided in Table 5. The performance of the CGD^24^ and ML-PC^14^ was employed as a benchmark to evaluate the DOT-AE-GAN. As anticipated, DOT-AE-GAN follows the lesion size measurements by ultrasound closely as compared with the CGD and ML-PC.^14^

**Table 5:** Quantitative comparison of reconstructed lesion diameter (cm) with clinical data.

US measured diameter	CGD, mean	ML-PC, mean	DOT-AE-GAN, mean
0.5 to 1.5	2.744 ± 0.648	1.196 ± 0.169	1.427 ± 0.396
1.5 to 2.5	2.665 ± 0.469	1.758 ± 0.195	1.874 ± 0.302
2.5 to 3.5	2.662 ± 0.525	1.675 ± 0.078	2.275 ± 0.544

## 6 Ablation Study

We conducted a series of ablation studies to evaluate the performance of different components of our model. Table 6 displays the results of the ablation study for different components of our model, for the maximum absorption coefficient in the reconstruction. The findings indicate that the model performance improved with respect to the error criteria when all three inputs—background absorption and scattering coefficients ($\mu_{a0}$ and $\mu_{s0}$), lesion location mask, and perturbation are incorporated into the design.

**Table 6:** Ablation study with quantitative analysis.

Ablation set	Mean absolute error	Mean square error
Perturbation only	0.2108	0.0476
Perturbation + mask	0.0964	0.0119
Perturbation + ($\mu_{a0},\mu_{s0}$)	0.0150	0.0033
Perturbation + mask + ($\mu_{a0},\mu_{s0}$)	0.0038	0.0001

We also conducted experiments to evaluate the impact of model parameters on performance. Our data show that DOT-AE-GAN outperforms the ML-PC model even when using similar layer types and maintaining a similar number of parameters. Notably, increasing the number of parameters in ML-PC^14^ leads to only marginal improvements in performance. To ensure a fair comparison, we excluded the discriminator parameters from this analysis. The quantitative results of this ablation study are presented in Table 7.

**Table 7:** Ablation study with model parameters and quantitative analysis.

Model	No. of parameters	Mean absolute error	Mean square error
ML-PC (FC layers only)	3,050,611	0.2501	0.0763
ML-PC (transformer-based)	12,470,979	0.2208	0.0494
DOT-AE-GAN	12,470,979	0.0038	0.0001

## 7 Summary

In this paper, we introduced DOT-AE-GAN, a hybrid model that integrates the advantages of autoencoders^19^ and GANs to tackle the challenges of lacking adequate training of irregularly shaped lesions encountered in US-guided DOT in breast patient studies. The autoencoder component of DOT-AE-GAN is designed to encode perturbation into reconstruction and decode reconstruction back into perturbation, effectively modeling both the inverse reconstruction process and the forward photon transport process. Simultaneously, the GAN component is incorporated to enhance AE reconstruction robustness through adversarial training from forward operator to inverse operator networks with irregularly shaped lesion inputs. We evaluated our approach using simulated, phantom, and clinical data, and comprehensive experiments demonstrate that DOT-AE-GAN performs better than regularized CGD in both maximum absorption coefficient and lesion size estimate. In addition, DOT-AE-GAN is more robust than ML-PC^14^ in lesion size estimates.

Although DOT-AE-GAN performs well in reconstructing both homogeneous and heterogeneous targets, the current model is limited to cases with single-target cases. Although preliminary tests suggest some ability to recover multiple closely spaced inclusions, reconstruction quality tends to decline as target complexity increases. To address these limitations, future work will focus on extending the model to handle multi-target configurations more effectively. In addition, our reconstruction framework currently assumes a smoothed absorption coefficient map modeled with a Gaussian distribution. Future research may explore alternative formulations to better represent diverse heterogeneous target profiles.

In conclusion, with the introduction of DOT-AE-GAN, we strive to optimize DOT image reconstruction regarding lesion size and reconstruction robustness. We believe that this hybrid model not only addresses current limitations but also opens new possibilities for applying advanced hybrid machine learning models to other challenging imaging problems.

## Data Availability

The code and data are available on GitHub: https://github.com/OpticalUltrasoundImaging/DOT-AE-GAN.git. Data underlying the results presented in this paper are not publicly available at this time but may be obtained from the authors upon reasonable request.
